# Atypical Features in a Large Turkish Family Affected with Friedreich Ataxia

**DOI:** 10.1155/2016/4515938

**Published:** 2016-09-07

**Authors:** Semiha Kurt, Betul Cevik, Durdane Aksoy, E. Irmak Sahbaz, Aslı Gundogdu Eken, A. Nazli Basak

**Affiliations:** ^1^Department of Neurology, Gaziosmanpasa University Faculty of Medicine, 60100 Tokat, Turkey; ^2^Suna and Inan Kıraç Foundation Neurodegeneration Research Laboratory, Molecular Biology and Genetics Department, Bogazici University, 34342 Istanbul, Turkey

## Abstract

Here, we describe the clinical features of several members of the same family diagnosed with Friedreich ataxia (FRDA) and cerebral lesions, demyelinating neuropathy, and late-age onset without a significant cardiac involvement and presenting with similar symptoms, although genetic testing was negative for the GAA repeat expansion in one patient of the family. The GAA repeat expansion in the frataxin gene was shown in all of the family members except in a young female patient. MRI revealed arachnoid cysts in two patients; MRI was consistent with both cavum septum pellucidum-cavum vergae and nodular signal intensity increase in one patient. EMG showed demyelinating sensorimotor polyneuropathy in another patient. The GAA expansion-negative 11-year-old female patient had mental-motor retardation, epilepsy, and ataxia. None of the patients had significant cardiac symptoms. Description of FRDA families with different ethnic backgrounds may assist in identifying possible phenotypic and genetic features of the disease. Furthermore, the genetic heterogeneity observed in this family draws attention to the difficulty of genetic counseling in an inbred population and to the need for genotyping all affected members before delivering comprehensive genetic counseling.

## 1. Introduction

Friedreich ataxia (FRDA) is an autosomal recessive neurodegenerative disorder and the most common hereditary ataxia. The FRDA locus was detected on chromosome 9 in 1988 [1]. The frataxin gene, which encodes a protein that probably acts as a mitochondrial iron transporter, was detected in 1996 [1]. Most mutations appear to be unstable expansions of a GAA repeat in the first intron of the gene [1].

Harding described the essential clinical features as (1) autosomal recessive inheritance, (2) onset before 25 years of age, (3) progressive limb and gait ataxia, (4) absent tendon reflexes in the lower extremities, (5) electrophysiological signs of axonal sensory neuropathy (within 5 years of onset), (6) dysarthria, (7) areflexia at all four extremities, (8) distal loss of position and vibration sense, (9) extensor plantar reflex, and (10) muscle weakness in the lower extremities [2]. The “typical” or “classic” form of the disease is defined as a disease form exhibiting all the clinical features listed by Harding [2, 3]. Disease forms which do not meet these criteria are referred to as atypical Friedreich ataxia. Skeletal deformities, cardiomyopathy, and diabetes are the most common systemic conditions associated with FRDA [4]. The disease is characterized by clinical variability regarding the age at onset, the rate of progression, and presence/absence of areflexia, muscle weakness, or cardiomyopathy. However, the clinical manifestations tend to be similar in affected siblings in the same family [4], indicating the crucial role of genetic factors for phenotypic expression of the disease.

In FRDA patients, magnetic resonance imaging scans reveal spinal cord atrophy along with normal brainstem, cerebellum, and cerebrum [2]. Demyelinating neuropathy and late disease onset after the age of 25 years are the manifestations excluding the classical form of FRDA [1, 3]. Cardiac involvement occurs in most of the patients with FRDA [2]. Similar symptoms related to multiple diseases are also rare in the same family [5].

Here, we describe the atypical clinical features of several members of the same family diagnosed with Friedreich ataxia (FRDA) and cerebral lesions, demyelinating neuropathy, and late-age onset without a significant cardiac involvement and presenting with similar symptoms, although genetic testing was negative for the GAA repeat expansion in one patient of the family.

## 2. Patients and Methods

Our index case was a 37-year-old man admitted to our outpatient clinic following a history of ataxia. Family members with similar symptoms were identified in Tokat, a city in the Middle Black Sea region of Turkey. This study was performed in accordance with the Helsinki Declaration. All adult participants provided written informed consent. Parents of participating minors provided written informed consent; literate minors who could write also provided signed assent. All subjects received a detailed explanation of the study and genetic counseling as appropriate. A pedigree was prepared in the field. A detailed history was obtained from each subject, and each subject received a detailed neurological examination. All available previous tests and imaging studies of the patients were recorded in detail.

Five stages of disease disability were distinguished: stage 0: patients at risk with normal examination results; stage 1: clinical signs of FRDA found at clinical exam; stage 2: functional signs but able to walk unaided; stage 3: clinical and functional signs, unable to walk without help; stage 4: confined to wheelchair, able to stand but not to walk; stage 5: bedridden: unable to stand [1].

*Electrophysiological Examination.* Electrophysiological studies were performed using standard nerve conduction techniques with Medelec-Oxford EMG equipment. Sensory nerve conduction studies were performed orthodromically on median and ulnar nerves and antidromically on the sural and superficial peroneal nerves. Motor nerve conduction studies were conducted on the median, ulnar, tibial, and peroneal nerves. Electromyography was performed on at least two proximal and distal muscles from the upper and lower extremities. 

*DNA Sample Collection and Genetic Analysis.* Blood samples were taken from the cubital vein into EDTA-containing tubes during field work. DNA was isolated using the Magna Pure Compact System from Roche, followed by PCR analysis.

## 3. Results

**Figure 1:**
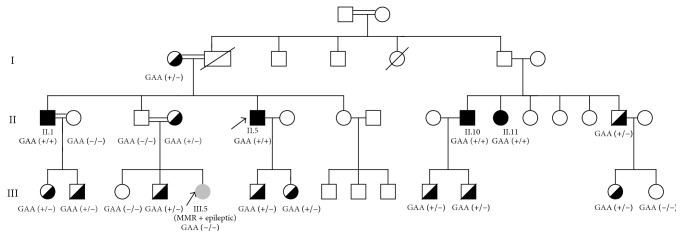
Pedigree of a family with FRDA. Black boxes indicate affected FRDA patients. The index case is indicated by an arrow. Semiblack boxes indicate FRDA carriers. The FRDA(−) case is indicated by grey box and the number III.5.

Figure 1 shows the pedigree structure of the family. Blood samples were obtained from five symptomatic and 15 asymptomatic family members during family screening. Neurological examination of 15 asymptomatic family members did not reveal any abnormal finding. Ten out of these fifteen family members were carriers of GAA repeat expansion. Clinical, electrophysiological, and neuroradiological data were obtained from two male and two female family members suffering from ataxia (Table 1). One male patient was evaluated clinically and genetically. Patients I.1 (the index patient) and II.5 were brothers, and Patient III.5 was their niece. Patients II.10 and II.11 were siblings and first cousins of Patients II.1 and II.5.

## 4. Clinical Presentation of the Family

### 4.1. Patient II.5 (Index Case)

**Figure 2:**
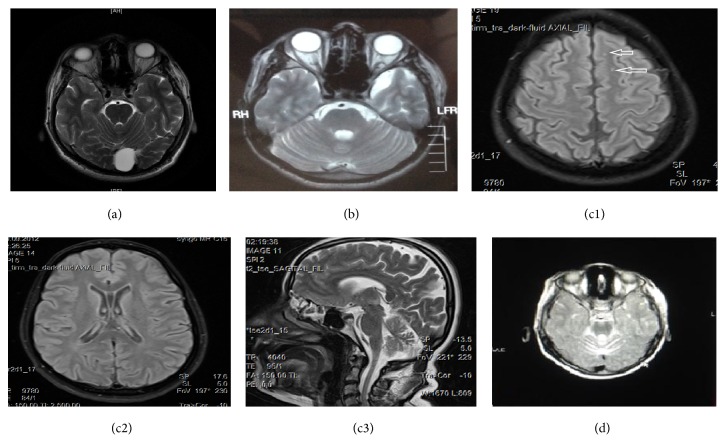
Arachnoid cyst in posterior vermian area in Patient II.5 (a); left temporal arachnoid cyst in Patient II.1 (b); multiple nodular (white arrows) lesions (c1), cavum septum pellucidum-cavum vergae variation (c2), and cerebellar atrophy in Patient II.11 (c3); cerebellar atrophy in Patient III.5 (d).

The 37-year-old male patient presented with a history of imbalance and numbness in hands and feet starting from the age of 18 without any other previous complaints. Although numbness in the hands and feet has improved in time, the imbalance had worsened. Following the increasing deterioration of the writing hand, muscle weakness in the hands and feet increased. Speech disturbance started about 6-7 years ago. 2-3 years ago, he started to require unilateral aid to walk due to the increased risk of fall. The patient defined similar complaints in his brother, in a female and male cousins, and in his niece. His neurological examination revealed dysarthric speech and a mild effacement of the left nasolabial fold, diffuse weakness and atrophy in the legs, and a mild distal atrophy in the hands. Deep tendon reflexes were absent in the upper and lower extremities. He had a moderate dysmetria and dysdiadochokinesia. His gait was ataxic in spite of unilateral walking aid. Joint-position sense was impaired in the toes. Plantar reflexes were absent on both sides. A mild thoracic scoliosis, pes cavus, and hammer toes were detected in his examination. The results of routine hematological and biochemical investigations were normal. He had no clinical symptoms of a cardiac disease and ECG was normal. In the electrophysiological studies, sensory action potentials were absent in the sural, superficial peroneal, median, and ulnar nerves. Median nerve conduction velocity (NCV) was 38.7 m/s, terminal latency (TL) was 4.3 ms, and* F* latency was 36.5 ms. Ulnar NCV was 33.45 m/s, TL was 4 ms, and* F* latency was 37.3 ms. Peroneal NCV was 27.3 m/s, TL was 6.2 ms, and* F* latency was 68.5 ms. Tibial NCV was 25.4 m/s, TL was 6.55 ms, and* F* latency was 64.4 ms. Conduction abnormalities matched demyelinating neuropathy criteria [6]. Brain MRI scans revealed an image of arachnoid cyst approximately 25 mm in diameter in posterior vermian area (Figure 2(a)).

### 4.2. Patient II.1

The 47-year-old male patient stated imbalance that started at the age of 27. Later on, speech disturbance was added to his complaints. Both imbalance and speech disturbance have gradually worsened and he was barely able to take a few steps with bilateral walking aid for the last 5-6 years, usually using a wheelchair. His neurological examination revealed dysarthric speech and bilateral horizontal nystagmus, a mild loss of muscle strength in the lower extremities in spite of normal muscle strength in the upper extremities, a normal superficial sensation and loss of vibration sense in the distal foot, absent deep tendon reflexes, and extensor plantar responses on both sides. Dysmetria and dysdiadochokinesia were detected in the upper limbs. He was barely able to stand and take a few steps. The results of routine hematological and biochemical investigations were normal. He had no clinical symptoms of cardiac disease and ECG was normal. Echocardiography revealed a mild diastolic dysfunction. On electrophysiological examination, sensory action potentials were absent in the sural nerve and moderately reduced in amplitude and velocity in the median and ulnar nerves. The median, ulnar, peroneal, and tibial motor conduction studies were normal.

Patient's previous MRI scans of the brain revealed a left temporal CSF-isointense lesion consistent with arachnoid cyst (Figure 2(b)) and previous MRI and X-ray of the thoracolumbar spine showed S-shaped scoliosis.

### 4.3. Patient II.10

The 52-year-old male patient stated gait imbalance and speech disturbance first started when he was sixteen. He became wheelchair-bound in 1997 due to gradually increased ataxia. His neurological examination revealed severe dysarthria, bilateral horizontal nystagmus, a normal superficial sensation, and reduced sense of vibration and position in the distal foot. There was no significant loss of muscle strength. Triceps and biceps reflexes were diminished while other deep tendon reflexes were absent too. Flexor plantar responses were obtained on both sides and severe bilateral dysmetria and dysdiadochokinesia were detected predominantly on the left side and he was unable to stand. Scoliosis was noted by visual inspection. The patient had no cardiac complaints and did not accept to undergo investigations other than genetic analysis.

### 4.4. Patient II.11

The 40-year-old female patient first started to have difficulty in walking when she was sixteen. The gait disorder was followed by a speech disturbance and loss of muscle strength particularly in the lower limbs, and she became wheelchair-bound at the age of 25. Her neurological examination revealed dysarthria, bilateral horizontal nystagmus, and muscle strength of 4/5 in the upper extremities and 0/5 in the lower limbs apart from muscle strength of 2/5 in the dorsiflexion and plantar flexion of the toes, on both sides. Muscle tone was reduced in the lower extremities. Vibration sense was reduced in the distal parts of the hands and foot, in addition to the glove-stocking hypoesthesia. Deep tendon reflexes were absent and an extensor plantar response was obtained on both sides. Severe bilateral dysmetria and dysdiadochokinesia were detected predominantly on the left side and she was unable to stand even with bilateral assistance. Moderate thoracic scoliosis was evident. The results of routine hematological and biochemical investigations were normal. She had no clinical symptoms of a heart disease and ECG was normal. On electrophysiological examination, sensory action potentials were absent in the sural, superficial peroneal, median, and ulnar nerves. The median, ulnar, peroneal, and tibial motor conduction studies were normal. MRI scan of the brain of the patient revealed signal changes of the white matter indicating multiple nodular lesions in the cerebral parenchyma at the level of corona radiata, centrum semiovale, a congenital cavum septum pellucidum, cavum vergae variation in the interventricular space, and cerebral and cerebellar atrophy (Figures 2(c1), 2(c2), and 2(c3)).

### 4.5. Patient III.5

The 11-year-old female patient with motor and mental retardation was born by Cesarean section for breech presentation at term. She kept her head steady at 8 months of age and sat without support at the age of three, crawled at the age of four and started to walk at the age of five. Subsequently, she has gradually developed a gait ataxia and required the assistance of a walker and then she became unable to walk. She had her first seizure when she was 6 months old. First valproic acid was started and then it was switched to lamotrigine. The seizures were taken under control by lamotrigine and the treatment was discontinued following two years of seizure-free period of time. It was reported that she was unable to talk in sentences but she could speak a few words and she was partly able to understand. In the neurological examination, she was capable of forming words in a dysarthric pattern. She was not oriented and poorly cooperative. Gross examination of the cranial nerves revealed no abnormality. Muscle strength was determined as 4/5 in the proximal parts of upper extremities and 3/5 in the distal parts and as 3/5 in the proximal parts of lower extremities and 0/5 in the distal parts. Muscle tone was reduced. Sensory and cerebellar tests could not be performed. Deep tendon reflexes were absent and plantar responses were extensor. Previous routine blood biochemistry and hematological tests were within normal limits. Amino acid levels were found to be within normal limits in an AA analysis using tandem MS (LC-MS-MS) method. C8/C12 ratio was found to be mildly elevated in the acylcarnitine/carnitine analysis; however, the result of the repeat test was normal. Serum levels of vitamin E, AFP, lactate, IgA, IgE, IgG, and IgM were found to be within normal levels. Urine organic acid assessment revealed an excretion of 50 mmol/mol of succinic acid. The excretion of oxalic acid was equal to the internal standard and the excretion of homovanillic acid and vanillylmandelic acid was equal to half of the internal standard along with elevated excretion of pyruvic acid, 3-OH isobutyric acid, ethylmalonic acid, adipic acid, 3-indol acetic acid, and 3-OH propionic acid and trace amount of erythro 4,5 diOH hexanoate lactone, tiglylglycine, and methyl citrate. The excretion of other organic acids was within normal limits. Increased SCA-specific CAG repeat was not found in any of the SCA 1, 2, 3, 6, and 7 loci. Personal-social development was determined at the level of 18.5 months of age, fine motor skills were found at the level of 14–19 months, language skills were found at the level of 22–30 months of age, and gross motor development was found to be at the level of 12 months in the Denver II developmental screening test. ECG and echocardiography tests were normal. Previous EEGs reported a slow background activity and sharp waves in the right temporooccipital regions and some of them were reported as normal. Motor nerve conduction studies were normal in the lower extremities and no response could be obtained from the sural nerve in the EMG which was interpreted as a technical issue. Cerebellar atrophy was reported in the MRI scan of the brain (Figure 2(d)). The repeat EEG was normal. Sensory nerve action potentials could not be obtained from the sural and superficial peroneal nerves and sensory nerve action potential amplitudes of the median and ulnar nerves were reduced in the repeat EMG study. Motor conduction studies were normal. The patient had scoliosis concave to the left.

## 5. DNA Analysis

PCR analysis for the GAA repeat in the frataxin gene revealed the homozygous presence of the GAA repeat in all four affected members of generation II. The young Patient III.5 did not have GAA repeat in this locus. The analysis of her parents validated the result, since her mother was a carrier, whereas the father was normal for the GAA repeat.

## 6. Discussion

In patients with Friedreich ataxia, MRI scans of the spine show thinning of the cervical spinal cord and signal abnormalities in the posterior and lateral columns [7]. Cerebellar atrophy is not a common finding in CT or MRI images; however, the presence of cerebellar atrophy indicates a severe, advanced disease [7, 8]. Brain MRI may be a useful diagnostic procedure, since the absence of cerebellar atrophy may point out other forms of hereditary recessive ataxia rather than Friedreich ataxia [2, 7]. However, as far as we know, no intracranial lesions have been reported in FRDA. Arachnoid cyst was reported in two of our patients and cavum septum pellucidum and cavum vergae variation and nodular signal changes were detected in Patient II.11. Arachnoid cysts and congenital intra-axial midline cysts (cavum septum pellucidum, cavum vergae, and cavum velum interpositum) are nonneoplastic neurological cysts [9]. Arachnoid cysts are fluid-filled duplications or splittings of the arachnoid layer with a content mildly different from the cerebrospinal fluid [10]. They may occur sporadically as isolated variations or may be associated with other malformations or diseases [10]. Usually arachnoid cysts are incidentally detected on imaging studies of the brain [11]. Although most cases are sporadic, intracranial arachnoid cysts in several members of the same family have been reported in a few publications [11]. In one of these reports, arachnoid cysts were accompanied by a deletion in chromosome 16 in the same family [11]. Jadeja and Grewal presented an unusual association of genetic myopathy, oculopharyngeal muscular dystrophy, and arachnoid cysts in the same family [12]. Değerliyurt et al. described two siblings with porencephaly, hemiparesis, epilepsy, and atrophic kidney associated with an arachnoid cyst in one of the siblings and in the asymptomatic mothers. Col4A1 gene mutation screening revealed a novel mutation in mother and both children [13]. Bayrakli et al. presented an intracranial arachnoid cyst family from southern Turkey with six out of seven offspring with intracranial arachnoid cysts in different localizations [14]. Arachnoid cyst was found to be present in two male siblings with FRDA in our study.

When a septum pellucidum has a separation between its two leaflets, it is referred to as cavum septum pellucidum (CSP). This condition takes place when there is separation between the leaflets of the septum pellucidum and posterior extension to the splenium of the corpus callosum. The anterior columns of the fornix separate the anterior cavum septum pellucidum and the posterior cavum vergae (CV). CSP may persist in up to 20% of adults. CV is present in up to 30% of newborns, although it may persist in less than 1% of the adult individuals. CV cysts are usually associated with CSP [9]. The CSP and CV cyst association was detected also in our patient.

Preadolescent onset is commonly regarded as crucial for the diagnosis of FRDA. Harding revised the diagnostic criteria in order to include patients with late onset up to the age of 25 years [3]. The complaints of Patient II.1 started after the age of 25. Late onset Friedreich ataxia, defined by symptom onset after the age of 25, accounts for 14% of the cases, while very late onset Friedreich ataxia, characterized by disease onset after the age of 40, is very rare [15].

Nerve conduction studies reveal axonal sensory neuropathy along with small or absent sensory action potentials in FRDA patients [2]. Motor conduction velocities are normal or mildly reduced in comparison to the sensory nerve potentials [2]. Nerve conduction studies of Patients II.1 and II.11 shared these characteristics; however, the motor nerve studies of our index case revealed demyelinating characteristics. According to Harding's criteria, a marked reduction of motor nerve conduction velocities is a finding that may exclude the diagnosis of FRDA [3]. However, Panas et al. reported three unrelated families with four affected children who suggested a hereditary motor and sensory neuropathy according to the clinical findings; however, the molecular genetic analysis was consistent with FRDA [16]. They claimed that the mutation identified in all four patients supports that these cases are representatives of a “variant” form of FRDA [16]. Similarly, Benomar et al. also reported that, in four out of seven FRDA patients, electromyography revealed a severe demyelinating neuropathy and severe demyelination and axonal neuropathy in the other three patients [1].

Cardiomyopathy is present in two-thirds of the patients with FRDA which is primarily symmetric concentric hypertrophic cardiomyopathy; in addition, some patients exhibit asymmetric septal hypertrophy [2]. Electrocardiogram reveals widespread T wave inversions and signs of ventricular hypertrophy [2]. The Acadian Type (Louisiana Form), which is observed in a specific population of French origin living in North America, was distinguished from typical FRDA by its milder course and lower incidence of cardiomyopathy [2]. Similarly to the Acadian Type, no significant cardiomyopathy was detected in our cases.

The EMG patterns and ages of onset were heterogeneous in our cases. The interfamilial clinical variability in FRDA patients was explained by mutation heterogeneity before the elucidation of the molecular basis of FRDA. The knowledge that almost all cases of FRDA are caused by the same dynamic mutation provided another way to interpret phenotypic heterogeneity [16]. According to Illarioshkin et al., the cooccurrence of distinct clinical variants of the disorder is associated with different combinations of the mutated alleles inherited from parents [4].

The genetic study of Patient III.5 was negative for FRDA. Bouhlal et al. have described three distinct gene defects leading to an autosomal recessive ataxia in a consanguineous Tunisian family [5]. A study conducted by Zlotogora asserted that a chance phenomenon, the migration of families with affected patients or a digenic inheritance, might be responsible for the genetic heterogeneity observed in some autosomal recessive disease. However, these explanations are not persuasive in most cases. Although the selection mechanism was demonstrated to explain most of the observations, it is difficult to prove [17]. The hypothesis of a coincidental association seemed to be the most logical explanation; however, it is also difficult to explain it on a statistical basis [5].

Description of FRDA families with different ethnic backgrounds may assist in identifying possible phenotypic and genetic features of the disease. Furthermore, the genetic heterogeneity observed in this family draws attention to the difficulty of genetic counseling in an inbred population and to the need for genotyping all affected members before delivering a comprehensive genetic counseling.

## Figures and Tables

**Table 1:** Clinical and laboratory characteristics of the patients.

Feature	Patient II.5	Patient II.1	Patient II.10	Patient II.11	Patient III.5
Age at evaluation	37	47	52	40	11
Age at onset	18	27	16	16	Infant
Duration of the disease	19	20	36	24	11
Sex	M	M	M	F	F
First symptom	Ataxia, paresthesia	Ataxia	Ataxia	Ataxia	MMR
Ataxia	++	+++	+++	+++	+++
Dysarthria	+	++	++	++	++
Muscle weakness					
Upper limbs	+	−	−	+	++
Lower limbs	+	+	−	+++	+++
Deep tendon reflexes					
Upper limbs	Absent	Absent	Diminished	Absent	Absent
Lower limbs	Absent	Absent	Absent	Absent	Absent
Joint position/vibration sense abnormalities					
Upper limbs	−	−	−	+	NP
Lower limbs	+	+	+	+	NP
Babinski sign	Absent	Extensor	Flexor	Extensor	Extensor
Extraneurological findings	Scoliosis, hammer toes	Scoliosis	Scoliosis	Scoliosis	Scoliosis
Cardiomyopathy	−	−	−	−	−
Cranial MRI	Arachnoid cyst	Arachnoid cyst	NP	CV, CSP, NL, and C-Ce atrophy	Ce-atrophy
NCS	Demyelinating neuropathy	Sensorial neuropathy	NP	Sensorial neuropathy	Sensorial neuropathy
Functional score	Unable to walk without help	Confined to wheelchair	Confined to wheelchair	Confined to wheelchair	Confined to wheelchair

M: male, F: female, MMR: mental motor retardation, NP: not performed, NL: nodular lesions, CV: cavum vergae, CSP: cavum septum pellucidum, C: cerebral, and Ce: cerebellar. +++: severe; ++: moderate; +: mild; −: no.
